# Thinking about Others’ Minds: Mental State Inference in Boys with Conduct Problems and Callous-Unemotional Traits

**DOI:** 10.1007/s10802-020-00664-1

**Published:** 2020-07-06

**Authors:** Ruth Roberts, Eamon McCrory, Geoffrey Bird, Molly Sharp, Linda Roberts, Essi Viding

**Affiliations:** 1 Division of Psychology and Language Sciences, University College London, 26 Bedford Way, London, WC1H 0AP UK; 2 Department of Experimental Psychology, University of Oxford, Oxford, OX1 3PS UK; 3 MRC Social, Genetic & Developmental Psychiatry Centre, Institute of Psychiatry, Psychology & Neuroscience, King’s College London, De Crespigny Park, Denmark Hill, London, SE5 8AF UK; 4 University of Manitoba, 66 Chancellors Cir, Winnipeg, Manitoba R3T 2N2 Canada

**Keywords:** Conduct problems, Callous-unemotional traits, Mentalising, Adolescent males

## Abstract

Children with conduct problems (CP) and high levels of callous-unemotional traits (CP/HCU) have been found to have an intact ability to represent other minds, however, they behave in ways that indicate a reduced propensity to consider other people’s thoughts and feelings. Here we report findings from three tasks assessing different aspects of mentalising in 81 boys aged 11–16 [Typically developing (TD) *n* = 27; CP/HCU *n* = 28; CP and low levels of callous-unemotional traits (CP/LCU) *n* = 26]. Participants completed the Movie Assessment of Social Cognition (MASC), a task assessing ability/propensity to incorporate judgements concerning an individual’s mind into mental state inference; provided a written description of a good friend to assess mind-mindedness; and completed the Social Judgement Task (SJT), a new measure assessing mentalising about antisocial actions. Boys with CP/HCU had more difficulty in accurately inferring others’ mental states in the MASC than TD and CP/LCU boys. There were no group differences in the number of mind-related comments as assessed by the mind-mindedness protocol or in responses to the SJT task. These findings suggest that although the ability to represent mental states is intact, CP/HCU boys are less likely to update mental state inferences as a function of different minds.

**Electronic supplementary material:**

The online version of this article (10.1007/s10802-020-00664-1) contains supplementary material, which is available to authorized users.

## Introduction

Children with conduct problems (CP) display a range of antisocial behaviours including bullying and manipulation, physical aggression, and violation of societal rules and norms (Frick 2016). Children with CP have a greater risk of physical and mental health problems, difficulties with personal relationships, as well as reduced employment and increased criminality in adulthood (Frick 2016; Rivenbark et al. 2018; Wertz et al. 2018). They require more support from specialist education provisions, have increased use of health and social care services, and increased contact with the criminal justice system which creates a significant financial burden for society (D’Amico et al. 2014; Frick 2016; Scott et al. 2001). This has created an impetus for earlier and more targeted intervention strategies to halt the development of CP for the good of the individual and society (Rivenbark et al. 2018; Stellwagen and Kerig 2013).

Considerable research has demonstrated that children with CP are a heterogeneous group and one way of understanding the heterogeneity of CP behaviours is to consider the role of callous-unemotional (CU) traits (Frick et al. 2014; Frick and Viding 2009; Viding and McCrory 2015). Children with CP and high levels of CU (CP/HCU) display a callous lack of remorse and guilt and marked deficits in empathy (Frick et al. 2014; Viding and McCrory 2015) and are thought to be at an increased risk of developing psychopathy in adulthood (Frick et al. 2014; Frick and Viding 2009). Children with CP/HCU not only display impulsive and reactive antisocial actions, but also commit calculated acts of aggression with little regard for other people’s feelings (Frick et al. 2014; Blair et al. 2014; Pardini and Byrd 2012). In contrast, children with CP and low levels of CU (CP/LCU) do not have pronounced deficits in empathy and remorse and often commit acts of aggression that have clear environmental triggers, such as perceived threat or frustration (Frick and Viding 2009; Blair et al. 2014). Measurement of CU traits (termed ‘Limited Prosocial Emotions’) was included in the latest edition of the Diagnostic and Statistical Manual of Mental Disorders 5th Edition (DSM-5) as a specifier for children with Conduct Disorder (American Psychiatric Association 2013).

A substantial amount of work has focussed on how children with CP/HCU process emotional signals from others. Children with CP/HCU have been found to have difficulties in processing emotional information, such as, having reduced neural responses when viewing other people in pain (Lockwood et al. 2013), reduced neural and behavioural responses to laughter (O’Nions et al. 2017), and difficulties in responding to and resonating with other people’s fear and sadness (Blair et al. 2014; Frick et al. 2014; Lozier et al. 2014; Viding et al. 2012). These difficulties, particularly difficulties with resonating with other people’s emotions, might in part explain why children with CP/HCU are able to engage in acts of aggression and violence and why they do not form typical affiliative relationships (Blair et al. 2014; Viding and McCrory 2019).

Another important aspect of social and emotional processing involves mentalising, which is the ability to understand the thoughts, intentions and feelings of other people (Fonagy and Allison 2012; Frith and Frith 2006). Mentalising is essential for all aspects of social interactions, allowing one to consider not only one’s own perspective, but also the various perspectives of others (Choudhury et al. 2006). Several studies have reported that children with CP/HCU are able to make accurate mental state inferences when the mentalising task does not require the participants to consider affective content (Anastassiou-Hadjicharalambous and Warden 2008; Jones et al. 2010; O’Nions et al. 2014; Schwenck et al. 2012). For example, Jones et al. (2010) found that CP/HCU children have difficulties with affective resonance, but not with cognitive perspective taking (i.e. mentalising without affective content), with the opposite pattern reported for children on the autism spectrum. Children with CP/LCU did not differ from TD peers on either affective resonance or cognitive perspective taking in this study. Other studies have reported similarly spared ability in making mental state inferences when children with CP/HCU are not required to mentalise about emotions (Anastassiou-Hadjicharalambous and Warden 2008; Schwenck et al. 2012). Additionally, O’Nions et al. (2014) reported that children with CP/HCU show recruitment of similar brain regions to TD peers when required to process scenarios that require mentalising, but which do not have affective content, whereas children on the autism spectrum show reduced activity in brain regions associated with mentalising compared with TD peers. This pattern of findings makes sense in the light of what is known about the behaviour of children with CP/HCU. They are able to successfully manipulate others for personal gain, which would not be possible without the ability to mentalise, however, they display clear deficits resonating with others’ feelings.

Although the basic ability to mentalise has been found to be intact, behaviours of children with CP/HCU suggest that they have a reduced propensity to mentalise (Viding and McCrory 2019). They tend to be more self-focused and can aggress even when someone is showing distress, especially if they stand to gain something (Jones et al. 2010; Pardini et al. 2003). A recent study by Drayton et al. (2018) has found that adult psychopaths can deliberately take the perspective of others, which may help them to manipulate others, but do not always spontaneously do so. Drayton et al. (2018) proposed that this pattern of functioning may enable individuals with psychopathy to avoid processing the emotional consequences of their antisocial behaviour towards other people or even orienting to other people’s needs in the first place. It seems that individuals with psychopathy can take on the perspective of others when it helps them achieve a goal but ignore it when it is not useful to them. In other words, part of the reason why individuals with psychopathy (or at risk of developing psychopathy) may so readily be able to prioritise ‘looking after number one’ could be due to their reduced tendency to consider other minds and/or make mental state inferences, while having the cognitive machinery to do so when it serves their own needs (Drayton et al. 2018).

The aim of the current study was to assess mentalising using three different tasks. We administered the Movie Assessment of Social Cognition (MASC) which asks participants to assess characters’ mental states after watching them interacting in a video (Dziobek et al. 2006). This task was selected as it presents a variety of information and cues (social, verbal, physical) and participants are asked to make assessments of thoughts, feelings, and intentions in ‘real-time’, similar to what one might encounter in real-life interactions with others (Sharp et al. 2011). It is thought to assess the ability/propensity to incorporate judgements about the protagonists’ minds into inferences about their mental states (e.g., whether one updates one’s estimate of the likelihood a character will be suspicious of another character based on whether the former character is thought to be paranoid or easy-going; Conway et al. 2019a, b). The MASC has previously been administered to a small sample of children with behaviour problems in a mainstream school (Körner et al. 2009). In this study, behaviour problems were associated with a reduced number of correct mentalising responses, however the rating of behaviour problems was limited to teacher reports on a single measure and no quantification of CU traits was provided (Körner et al. 2009). We also asked children to complete a standardised mind-mindedness task, which assesses the tendency to think about the minds of peers that are relevant to the participant. This task requires the participants to spontaneously describe a good friend, with no restrictions or limitations on their description. Previous research has found that young adults are more likely to make mind-related comments about someone they know personally, rather than a stranger, with intimacy providing greater knowledge of and ease of access to the person’s mental states (Meins et al. 2014). Children with CP/HCU may not be motivated to mentalise about strangers unless they can personally gain something out of it, however it may be less effortful and more instrumentally useful for them to consider the minds of peers that they regularly interact with. Finally, we administered the Social Judgement Task (SJT), an illustrated mentalising task that asked the participants to report what other children would think about them, if they engaged in a negative interaction with a fictional peer. The negative interaction scenarios in the SJT were developed to assess whether acting antisocially may be, in part, explained by difficulty in accurately predicting how the antisocial acts are viewed by others. This task provides insight into whether children with CP/HCU can infer what other people, specifically peers, think when they engage in social transgressions against others. The participants are also asked to report on the likelihood of committing acts described in the scenarios, providing a possible index of acting antisocially, despite knowing how it is viewed by others.

We chose to focus on groups of boys with CP/HCU and CP/LCU instead of conducting continuous analyses for the following reasons: 1) Effects of having distinct subgroups of children with CP as divided on CU traits do not often emerge as interactions and can instead lead to suppressor effects in correlational analyses (Frick 2012); 2) We know that bivariate normality does not apply to CP and CU distributions where high CU traits almost invariably denote high levels of CP, but not the other way around (Fontaine et al. 2011). Dichotomizing leads to reduction of power in the case of bivariate normality (Cohen 1983), but we know that bivariate normality does not apply to CU traits and CP; 3) The median split approach has, in the past, successfully delineated groups of children with CP who have different cognitive-affective processing patterns – often in a manner that would lead to the two groups cancelling each other out if pooled into a single CP group for comparison with typically developing children, or which do not necessarily emerge in dimensional analysis in community samples that represent the whole spectrum of scores. The child/group centric analyses also make it easier to interpret the translational relevance of findings, which is more challenging when examining potential suppressor effects. Some previous cross-sectional research has found higher mean levels of CU traits in older adolescents (Essau et al. 2006), although this is not evident in longitudinal data (Pardini and Loeber 2008). However, to ensure that age differences were not accounting for the findings, the groups were matched on age, with comparable representation across the age bands of the sample.

Although experimental findings indicate that individuals with or at risk of developing psychopathy have an intact ability to represent other minds (Anastassiou-Hadjicharalambous and Warden 2008; Jones et al. 2010; O’Nions et al. 2014; Schwenck et al. 2012), their behaviour suggests a reduced propensity to consider others. At the cognitive level this may manifest as: i) a reduced ability/propensity to incorporate mind type into mental state inference (as assessed by the MASC); ii) a reduced propensity to represent the minds of others (as indexed by the Mind Mindedness task); or iii) reduced ability to infer what other people think about them when they engage in social transgressions (as assessed by the SJT). The purpose of this study was to investigate each of these possibilities.

## Method

### Participants

Families were recruited from the community in the greater London area, via newspaper advertisements and from mainstream schools and schools who provide alternative education for children with behavioural difficulties. One hundred and fifty-eight families were screened for participation. Sixty-nine families did not participate (57 did not meet study criteria; 6 CP and 3 TD families had scheduling problems; 1 CP and 2 TD children refused to participate). Eighty-nine families were included in the main study, however, 8 children refused to take part in the mentalising tasks which left a total of 81 boys (aged 11–16 years) in the study. There was no significant difference in child age between participants and non-participants (age obtained at screening), *t* (156) = 1.172, *p* = 0.243. Participant characteristics are displayed in Table 1. The research was approved by the University College London Research Ethics Committee (Project ID number: 0622/001). Parents/caregivers and the boys were provided with information sheets outlining the details of the study and were given an opportunity to ask questions and seek clarification regarding their participation. Parents/caregivers provided written informed consent and written assent to participate was obtained from all boys. An experienced clinician provided training to researchers beforehand on how to sensitively work with boys with CP and their families. Exclusion criteria for child participants included a formal diagnosis of autism spectrum disorder, any reported neurological disorder, use of prescription medication for behavioural difficulties, and cognitive ability of <70 on a standardised cognitive assessment. Parent/caregivers were not subjected to any exclusion criteria. All families were provided with a £50 honorarium to cover travel expenses and lunch.

**Table 1:** Demographic data

	TD controls (*n* = 27)			CP/LCU (*n* = 26)			CP/HCU (*n* = 28)				
Characteristics and questionnaires	Mean	S.D.	(Min-Max)	Mean	S.D.	(Min-Max)	Mean	S.D.	(Min-Max)	*p* value ^a^	Post hoc*
Child age (years) ^b^	14.37	1.43	(11–16)	14.57	1.65	(11–16)	14.71	1.23	(12–16)	0.678	
Child IQ (full score, two-subtest WASI) ^c^	91.48	11.45	(72–122)	93.46	12.63	(70–118)	88.70	11.11	(76–113)	0.338	
Child ethnicity ^b,f^	16:4:7			8:4:14			20:3:5			0.033	
SES ^b^	2.84	1.22	(1–5.5)	3.12	1.18	(1.25–5)	3.38	1.17	(1.5–5.5)	0.079	
ICU ^d^	25.74	6.04	(13–38)	33.46	6.76	(15–42)	49.32	5.68	(43–63)	0.000	1 < 2 < 3
CASI Conduct disorder ^d^	0.78	0.75	(0–2)	6.12	2.88	(3–15)	13.36	6.52	(4–31)	0.000	1 < 2 < 3
CASI Attention deficit hyperactivity disorder ^e^	12.4	9.36	(1–38)	24.17	12.27	(2–47)	28.56	12.55	(6–52)	0.000	1 < 2/3
CASI Generalised anxiety disorder ^e^	4.77	4.49	(0–18)	6.81	3.86	(0–15)	10.19	4.56	(1–19)	0.000	1/2 < 3
CASI Major depressive episode ^e^	3.48	2.41	(2–13)	5.69	4.27	(2–17)	8.46	4.61	(2–19)	0.000	1/2 < 3
(BES) Cognitive empathy ^c^	35.55	3.08	(29–39)	35.12	4.70	(23–45)	32.85	5.22	(16–41)	0.061	
(BES) Affective empathy ^c^	34.98	4.78	(27–46)	34.16	7.68	(20–49)	29.40	5.31	(22–41)	0.001	1/2 < 3
**(**IRI – PT**)** Perspective taking ^c^	14.96	4.14	(5–22)	13.85	5.64	(4–25)	10.89	4.90	(3–22)	0.009	1 > 3
**(**AQC**)** Alexithymia ^c^	15.37	6.53	(4–27)	15.49	6.63	(3–28)	14.95	6.81	(2–28)	0.952	

*TD*, typically developing; *CP/LCU*, conduct problems and low levels of callous-unemotional traits; *CP/HCU*, conduct problems and high levels of callous-unemotional traits; *S.D.*, standard deviation; *WASI*, Weschler Abbreviated Scale of Intelligence; *SES*, socio-economic status; *ICU*, Inventory of Callous-Unemotional Traits; *CASI*, Child and Adolescent Symptom Inventory; *BES*, Basic Empathy Scale; *IRI-PT*, Perspective taking subscale of the Interpersonal Reactivity Index; *AQC*, Alexithymia Questionnaire for Children *p < 0.05, Games

-Howell post hoc comparison

a All *p* values obtained using Welch ANOVA, except child ethnicity (Chi-square)

b Measures obtained at screening phase, parent report

c Measures obtained at testing session, child report

d Measures obtained at screening phase, parent and teacher report

e Measures obtained at testing session, parent report

f White:Black:Mixed/Other

### Measures

#### Screening

Screening questionnaires assessing CP, CU traits, and psychopathology were completed by parents/caregivers and teachers to determine CP/HCU, CP/LCU, and TD groups prior to participation. Screening measures were scored by taking the highest ratings from either the parent or teacher questionnaire for each item (Piacentini et al. 1992). There was a statistically significant, moderate, positive correlation between parent and teacher ratings of CP (CP: *r*_*s*_ (68) = 0.42, *p* < 0.001) and CU (CU: *r*_*s*_ (68) = 0.49, *p* < 0.001). Teacher ratings were unavailable for five boys with CP/HCU, seven boys with CP/LCU and two TD boys.

CP was assessed using the *Child and Adolescent Symptom Inventory* (CASI-4R; Gadow and Sprafkin 2009) Conduct Disorder scale (CASI-CD), a widely used measure demonstrating good reliability and validity (Sprafkin et al. 2002). In our sample, the CASI-CD had a good level of internal consistency as determined by a Cronbach’s alpha of 0.83. Inclusion for the CP group required that the CASI-CD score met either parent or teacher severity cut-off (parent report = 4+ (ages 10–12) and 3+ (ages 13–16) or teacher report = 3+ (ages 10–12), 4+ (ages 13–14), and 6+ (ages 15–16)). These scores are associated with a clinical diagnosis of conduct disorder (Gadow and Sprafkin 1998). Fifty-four boys meeting the screening criteria for CP were recruited for this study.

CU traits were assessed using the *Inventory of Callous-Unemotional Traits,* which has been found to have good reliability and validity (ICU; Essau et al. 2006). In our sample, the ICU had a high level of internal consistency as determined by a Cronbach’s alpha of 0.92. Boys meeting CP criteria were assigned to CP/HCU and CP/LCU groups based on a median split of the ICU scores. Twenty-six boys met CP/LCU criteria with ICU scores less than or equal to 42 and twenty-eight boys met criteria for CP/HCU with ICU scores greater than 42. Other studies employing the median split approach to assign boys with conduct problems into CP/HCU and CP/LCU groups have reported median scores of the ICU ranging from 30 to 42 (Hodsoll et al. 2014; Jones et al. 2010; Martin-Key et al. 2017; O’Nions et al. 2017; Roberts et al. 2018; Schwenck et al. 2012; Sebastian et al. 2016; Sethi et al. 2018) and a recent study has suggested that a score of 41 may represent a clinically meaningful cut-off for HCU (Docherty et al. 2017). The median split of 42 in the current study is thus higher than or comparable to median split scores in previous research and designates a group of children with extreme CU scores within clinically significant range (estimated to be within the top 5% of the population).

The *Strengths and Difficulties Questionnaire* (SDQ; Goodman 1997) was used to screen for emotional and behavioural difficulties in the control participants. Twenty-seven boys met screening criteria for inclusion in the TD group, scoring ≤2 on the CASI-CD, ≤38 on CU traits, and less than 17 on the SDQ Total Difficulties subscale (outside the abnormal range as per SDQ scoring norms; Youth in Mind 2016), not meeting exclusion criteria.

#### Movie Assessment of Social Cognition (MASC; Dziobek et al. 2006)

The MASC is a video-based assessment of mentalising. Participants viewed four characters (young adults, two males and two females, from White ethnic backgrounds) making arrangements to meet up for dinner. The video is divided into short segments and at the end of each segment participants were presented with a multiple-choice question asking them to infer the mental state of one of the characters. The task required participants to attend to verbal, social, and physical cues from the characters as one might typically do in real-life interactions (Sharp et al. 2011). The video was presented on a Dell laptop using Psychopy software (Peirce 2007). Participants selected one of four response options on the computer keypad and were given as much time as needed to consider their response. In line with previous studies (Feyerabend et al. 2018; Newbury-Helps et al. 2017), items were grouped into questions that assessed characters ‘intentions’ or cognitive mentalising (e.g. Why is Sandra saying this?; nine items) and questions that assessed the ‘feelings’ of characters or affective mentalising (e.g. What is Betty feeling?; eight items). Three control questions asking participants about details of the scene (e.g. How many adults were in the scene?) were also included to ensure that participants paid attention to the task.

As it is difficult to keep children with CP engaged in lengthy assessments, the original task was shortened from forty-five questions (plus 5 non-social control questions) to 17 questions (plus 3 non-social control questions). This decision was made based on analysis of a large corpus of published and unpublished data indicating that the total score after 17 questions was correlated approximately 0.8 with the total score based on 45 questions (Shah et al. 2017). In this sample, Cronbach’s alpha for the shortened version of the task was 0.62.

#### Mind-Mindedness (Meins and Fernyhough 2015)

Mind-mindedness was assessed via participants’ hand-written descriptions of a person they considered to be a good friend (Meins et al. 2006). Using methods developed for older children, participants were asked to describe their close friend with open-ended, written responses to the following question: *Please describe your good friend - no specific type of description is required, you should just write whatever comes into your head* (Meins et al. 2006, 2008)*.* Participants were not restricted in the length of their description or the time it took to complete their response. The text was divided into segments and coded using the following exhaustive and exclusive categories: *Mind-minded* (referencing feelings, emotions, intellect, or mental states of the person being described); *Behavioural* (referencing activities, behaviours, or interactions that were behavioural in nature); *Physical* (referencing physical attributes, including age); and *General* (comments not belonging to any of the previous categories, such as length of friendship; or ambiguous statements, such as: ‘he’s great’) as detailed in the Mind-Mindedness Coding Manual (Meins and Fernyhough 2015). Higher numbers of mind-related comments indicated greater mind-mindedness. Although mind-minded descriptions are not typically analysed for affective content, for the purposes of this paper, mind-minded comments were further categorised as being affective if they were referencing their friend’s feelings or emotions.

#### Social Judgement Task (SJT)

The SJT is a cartoon measure assessing child perception of their peers’ point of view about antisocial interactions. Participants were presented with a series of five illustrated stories and asked to imagine that they have engaged in an instrumental antisocial interaction with a fictional peer (all fictional peers were depicted as adolescent males from Black and White ethnic backgrounds). They were given three multiple choice options: (1) other children would find the interaction acceptable, (2) other children would find the interaction unacceptable, or (3) a socially naive response not focussed on the interaction. Participants were specifically directed to mentalise in this task, by imagining themselves as the main character in the story and then thinking about what their peers would think about them following the interaction. The task was not designed to assess any affective aspects of mentalising (participants were asked what peers would think about them, rather than how peers would feel about them). The five antisocial scenarios were presented alongside ‘filler scenarios’ (three positive and two neutral scenarios) in a pseudorandomised order. The ‘filler scenarios’ were included to avoid the possibility of participants making automated computations about social norms, so that the participants had to consider each scenario individually. However, the antisocial scenarios were the focus of this task. This task was validated on a sample of 186 children from a mainstream secondary school in the Greater London area. The antisocial interactions were found to have good internal consistency (α = 0.81) and good construct validity as demonstrated by correlations between ‘belief that peers would say negative interactions are acceptable’ and CP (*r*_*s*_ (178) = 0.18, *p* < 0.05) and CU (*r*_*s*_ (175) = 0.24, *p* < 0.001). Full details of the development and validation of the SJT and an example of an antisocial scenario can be found in Online resource 1.

#### Additional Measures

Boys completed the *Wechsler Abbreviated Scale of Intelligence* (WASI; Wechsler 1999) two-subtest version to assess cognitive ability. Parents/caregivers provided information about parental education (scored using the six output categories for educational attainment from the Office of National Statistics 2004) and employment (scored using the Office of National Statistics occupational coding tool: https://onsdigital.github.io/dp-classification-tools/standard-occupational-classification/ONS_SOC_occupation_coding_tool.html; Office for National Statistics 2020) to determine family socio-economic status (SES). Parents/caregivers completed the CASI-4R scales for attention deficit hyperactivity disorder (ADHD), generalised anxiety disorder (GAD), and major depressive episode (MDE) to assess for commonly occurring comorbidities with CP. The CASI-4R subscales were found to have good internal consistency in this sample (CASI-ADHD α = 0.96; CASI-GAD α = 0.86; CASI-MDE α = 0.84). To assess features that might explain mentalising differences between groups, we obtained participants’ self-reported affective and cognitive empathy using the Basic Empathy Scale (BES; Jolliffe and Farrington 2006), perspective taking using items from the Interpersonal Reactivity Index (IRI-PT; Davis 1980), and alexithymic traits using the Alexithymia Questionnaire for Children (AQC; Rieffe et al. 2006). Good internal consistency was found for the BES, IRI-PT and AQC in this sample (BES affective α = 0.82; BES cognitive α = 0.79; IRI–PT α = 0.76; AQC α = 0.77). See Table 1 for details of the measures reported here.

### Procedure

Participants completed all assessments in a quiet testing room at University College London. Participants completed assessments independently from their parents/caregivers to ensure their responses were confidential. As child participants could not be left unattended, a researcher was on hand to answer questions. The researcher monitored compliance on all tasks. Participants watched the video using noise cancelling headphones to help minimise distraction.

### Statistics

#### Demographics

To examine the demographic characteristics of the groups, a one-way Welch ANOVA was computed to compare differences between the means for age, IQ, traits, CASI CD, ADHD, GAD, and MDE subscales, BES, IRI-PT, AQC, and family SES. Games-Howell post hoc analyses were conducted to examine differences between groups on the demographic variables. Chi-square was computed to compare groups on ethnicity. To further examine age matching within the three groups of participants, age was grouped into three bands: 11–12 years (TD *n* = 4; CP/LCU *n* = 5, CP/HCU *n* = 2), 13–14 years (TD *n* = 12; CP/LCU *n* = 10, CP/HCU *n* = 12), and 15–16 years (TD *n* = 11; CP/LCU *n* = 11, CP/HCU *n* = 14). Chi-square was computed to compare groups on the three age bands. A one-way Welch ANOVA was computed to compare differences in mean CU scores for the three age bands.

#### MASC

A one-way ANOVA was computed to determine if the groups differed on the ‘feelings’ (affective mentalising) and ‘intentions’ (cognitive mentalising) questions and control questions. Where overall significant group differences were found, Tukey’s post hoc analyses were computed to examine the differences between groups. Cohen’s d was computed to quantify the difference between the groups. Analysis of covariance (ANCOVA) was computed to control for all variables that were correlated with MASC performance or group status, which included ADHD, GAD, MDE, BES cognitive, BES affective, and IRI-PT.

#### Mind-Mindedness

Prior to conducting analysis, data entry was checked for accuracy and completeness. Any identifiable information was removed. Data was segmented into statements prior to coding. The entire data set was double coded by two raters who were masked to the participant group status. Cohen’s Kappa revealed a ‘substantial’ agreement between raters, κ = 0.824 (Landis and Koch 1977). To control for group differences in verbosity, scores were computed as a percentage of the total number of statements (Meins and Fernyhough 2015). A one-way ANOVA was computed to determine if the groups differed on any of the coding categories, as well as the number of affective mind-minded comments. Pearson chi-square was computed to determine if the groups differed on the percentage of mind-minded descriptions.

#### SJT

The number of acceptable, not acceptable and neutral responses to the five antisocial scenarios was computed for each participant. The scores for each scenario were binomially distributed (e.g. acceptable or not; not acceptable or not; neutral or not) so a generalized linear model was computed to ascertain the effect of group on the odds of thinking that other children would find the behaviour acceptable, unacceptable, or neutral. The generalised linear model assumes the odds of a subject saying something is acceptable (or not acceptable, or neutral) is the same across all five scenarios. To check this assumption, Fisher’s exact tests were computed to see if the groups differed on responding to any of the individual scenarios. ANOVA was computed to determine if the groups differed in terms of their likelihood of committing the described antisocial interactions.

## Results

### Demographics

Demographic information is presented in Table 1. No differences were found between groups on age, IQ, or SES. The groups differed on ethnicity, with the CP/LCU having fewer boys from white backgrounds and more boys from mixed ethnic backgrounds than the TD and CP/HCU groups. The CP/HCU and CP/LCU had significantly higher ADHD scores than the TD group, but the two CP groups did not differ significantly from each other. The CP/HCU group had significantly higher levels of anxiety and depression than the TD and CP/LCU groups who did not differ on anxiety and depression. The CP/HCU group had significantly lower levels of affective empathy (as measured by the BES) and perspective taking (as measured by the IRI-PT) than the TD and CP/LCU groups, who did not differ on these measures. There were no statistically significant group differences on cognitive empathy (as measured by the BES) or alexithymia (as measured by the AQC). However, the group difference in the cognitive empathy score (as measured by the BES) did approach significance (*p* < 0.06) and CP/HCU had the lowest level of cognitive empathy across the groups. No differences were found between groups on the three age bands (i.e. 11–12 years, 13–14 years, 15–16 years) *X*^*2*^(4) = 1.92, *p* = 0.75. There were no significant differences in CU scores across the three age bands *F* (2, 78) = 1.48, *p* = 0.233.

### MASC

#### “Intentions” vs “Feelings”

There was an overall group difference on the mean proportion of correctly identified ‘intentions’ questions, *F* (2, 78) = 5.448, *p* = 0.006, (TD *M* = 0.70; CP/LCU *M* = 0.67; CP/HCU *M* = 0.52). Post hoc analysis revealed statistically significant differences between CP/HCU and TD groups, with a large effect size (*p* = 0.009; *d* = 0.853) and between CP/HCU and CP/LCU groups, with medium effect size (*p* = 0.029; *d* = 0.671). The groups did not differ significantly on the ‘feelings’ questions, *F* (2, 78) = 0.737, *p* = 0.482, (TD *M* = 0.57; CP/LCU *M* = 0.56; CP/HCU *M* = 0.52), but it appeared that all groups struggled with the ‘feelings’ items, ranging from 52 to 57% correct on these items.

#### Control Questions

The three groups did not differ significantly on the three control questions, *F* (2, 78) = 0.75, *p* = 0.475. All groups performed well on the control questions (TD *M* = 2.70; CP/LCU *M* = 2.54; CP/HCU *M* = 2.50) indicating good attention to the task.

#### Covariate Analysis

We examined how group membership, child characteristics and task performance related to each other using Spearman’s Rho correlation analysis (See electronic supplementary material Table 1). We then ran an ANCOVA entering all of the child variables that correlated with the MASC performance or group status (ADHD, GAD, MDE, BES cognitive, BES affective and IRI-PT). This analysis showed that the effect of group on MASC ‘intentions’ was no longer statistically significant after adjusting for BES cognitive *F* (2, 76) = 2.879, *p* = 0.062*.*

### Mind-Mindedness

There was an overall group difference on total number of statements, *F* (2, 78) = 3.358, *p* = 0.040, (TD *M* = 4.52; CP/LCU *M* = 3.43; CP/HCU *M* = 3.54), however post hoc analyses revealed no significant difference between any of the three groups. To control for verbosity, scores for each category were computed as a percentage of the total number of statements. No differences were found between groups on mind-minded descriptions of close friends, *F* (2, 78) = 1.063, *p* = 0.351; on affective mind-minded descriptions, *F* (2, 57) = 0.447, *p* = 0.642; on behavioural descriptions, *F* (2, 78) = 0.838, *p* = 0.436; on physical descriptions, *F* (2, 78) = 2.557, *p* = 0.084; and on general descriptions, *F* (2, 78) = 0.899, *p* = 0.411. No differences were found between groups on the number of participants who generated no mind-minded descriptions of their friend, *X*^*2*^(2) = 2.906, *p* = 0.234.

### SJT

Results did not reveal any group differences for any of the individual antisocial interaction scenarios (Table 2), which meant that we were able to group these items for analysis. As detailed in Table 3, there was no effect of group on responses to the antisocial interaction scenarios (i.e. group was not affecting the likelihood of indicating that peers would find the antisocial scenario acceptable, unacceptable, or neutral). Groups did not differ on likelihood of committing the described negative actions, *F* (2, 75) = 1.845, *p* = 0.165, (TD *M* = 8.148; CP/LCU *M* = 9.76; CP/HCU *M* = 10.15).

**Table 2:** Fisher’s exact (and item counts) for acceptable, not acceptable, and neutral responses on the five ‘negative’ SJT scenarios by group

	Scenario one (Queue)			Scenario two (Crisps)			Scenario three (Art)			Scenario Four (Fun fair)			Scenario five(Treats)		
	Acceptable	Not Acceptable	Neutral	Acceptable	Not Acceptable	Neutral	Acceptable	Not Acceptable	Neutral	Acceptable	Not Acceptable	Neutral	Acceptable	Not Acceptable	Neutral
TD	2	17	7	12	14	0	4	21	2	12	11	4	2	21	4
CP/LCU	2	19	3	7	15	3	4	19	2	16	9	0	3	21	1
CP/HCU	2	22	3	11	13	3	5	20	2	17	9	1	1	24	2
	Total = 77	*p* = 0.163		Total = 78	*p* = 0.307		Total = 79	*p* = 1.00		Total = 79	*p* = 0.235		Total = 79	*p* = 0.575

**Table 3:** Generalised linear model predicting likelihood of beliefs about SJT negative interaction scenarios

	*Wald*	*df*	*p*
Peers would say acceptable ^a^	0.150	2	0.928
Peers would say unacceptable ^b^	0.076	2	0.963
Peers would say neutral ^c^	2.979	2	0.232

^1^Goodness of fit (Pearson’s *X*^2^ (a: 1.109; b: 1.176; c: 1.141)) did not indicate over dispersion

^2^As the distribution of responses was different for scenarios two and four as compared to scenarios one, three, and five (see Table 2), the analysis was repeated excluding scenarios two and four. This did not change the findings; no effect of group on responding (acceptable, unacceptable, or neutral) was found

## Discussion

Boys with CP/HCU had difficulty mentalising (as compared with TD and CP/LCU boys) when they performed a complex, ecologically valid task which indexed the ability/propensity to incorporate judgements about another’s mind type into inferences about their mental state (the MASC task). However, they did not differ from TD boys in their propensity to represent the minds of their friends when asked to describe them, or in their ability to understand that other children would think negatively about someone committing antisocial acts*.* Boys with CP/LCU did not differ from TD boys on performance in any of the three tasks. These findings provide a more nuanced picture of mentalising in boys with CP. Overall, they are in line with prior studies suggesting an intact ability to mentalise in children with CP, including those with CP/HCU, especially if there is no requirement to consider other people’s feelings. These findings also suggest that despite having the ability, boys with CP/HCU may have a reduced propensity to mentalise than their peers. They may only deploy this ability spontaneously if it does not require them to process complex information or if it is of instrumental benefit to themselves.

In line with our hypotheses, boys with CP/HCU had difficulty with the MASC task, in particular with the ‘intentions’ questions (assessing cognitive mentalising). MASC, unlike most assessments of mentalising, depicts people interacting in real life situations. Task performance depends on the ability/propensity to incorporate information about each character’s mind in order to make accurate mental state inferences during an observed ‘live’ interaction (Conway et al. 2019b; Dziobek et al. 2006). The effect of group on the ‘intentions’ questions was no longer significant after adjusting for cognitive empathy (as measured by the BES cognitive scale). Although the groups only showed a trend level difference on BES cognitive empathy, the CP/HCU boys had the lowest scores on this measure and the BES cognitive empathy scale taps into ability/propensity to incorporate information about other people’s minds to make accurate mental state inferences. It therefore follows that cognitive empathy would be having effect on correct responding to ‘intentions’ or cognitive items in the MASC as both are focussed on understanding the perspective of others. CP/HCU children may not be interested in others’ minds unless other people are instrumentally valuable, or they have a mind that is vulnerable or easy to manipulate. It could also be that the characteristics of children with CP/HCU mean that they will experience a restricted range of social interactions with other people, which may in turn reduce the number of types of mind to which CP/HCU children are exposed. While CP/HCU boys had clear difficulties with the ‘intentions’ questions in the MASC, they did not significantly differ from TD or CP/LCU participants in spontaneously mentalising about ‘feelings’ (affective mentalising). Although this may seem surprising, it is important to note that all groups had difficulties with the ‘feelings’ questions and it is likely that no group differences emerged because of a floor effect. It would, therefore, be inappropriate to conclude that boys with CP/HCU do well in spontaneously mentalising about feelings (in fact their rate of mentalising about feelings was very similar to their rate of mentalising about intentions). Instead, it appears that adolescent boys from similar SES backgrounds and of similar cognitive ability all show low levels of spontaneous mentalising about emotions.

Boys with CP/HCU showed reduced spontaneous mentalising about the interactions of strangers in the MASC task, but there were no group differences when boys were asked to spontaneously mentalise about a friend. CP/HCU boys appear similar to CP/LCU and TD peers in their propensity to represent friends’ minds. This may be explained by the greater knowledge one has about friends rather than someone with whom there is no personal relationship (Meins et al. 2014). Familiarity makes it easier to represent the mental states of friends. CP/HCU boys may also be more motivated to represent the minds of friends, as understanding friends’ point of view could be instrumentally valuable, if for no other reason than for successful manipulation. It may also be that CP/HCU have a similar mind type to their friends which makes it easier to infer mental states (Conway et al. 2019b). There were no group differences on affective mind-minded comments, but as was found with the MASC where all groups had difficulty with the ‘feelings’ questions, all groups had low levels of mentalising about their friends’ feelings and emotions. It would, therefore, be inaccurate to conclude that CP/HCU boys are inclined to consider their friends feelings when describing them.

Although the CP/HCU group had difficulty with the MASC task, they had an intact ability to infer the thoughts of others regarding engagement in antisocial actions. Boys with CP/HCU knew just as well as typically developing boys that peers would find antisocial acts unacceptable. This indicates that they can understand what is wrong and more critically how that is perceived by their peers. The SJT task does not require any inference of others’ feelings and it may be helpful for future research studies to include an affective component to explore whether group differences occur when children are asked how they might feel if they acted as the antisocial story described or how peers would feel about them if they acted antisocially. Interestingly, CP/HCU boys were not more likely to say they would act antisocially, as described in the story, than their TD or CP/LCU peers. It is instrumentally valuable to consider the thoughts of others with regard to antisocial actions and only execute such actions when the outcome is judged to be sufficiently valuable to discard the displeasure of others. In this case, it may not have been worth discarding the potential displeasure of the researcher given that there was nothing tangible to be gained by reporting that they would be likely to act as the story described. It is not adaptive to act in an antisocial way at all times, as this is likely to preclude taking maximal advantage of someone.

## Limitations

A number of limitations should be noted. There is a need to extend the study of spontaneous mentalising in CP/HCU in several ways. We currently have a poor understanding of factors that may impact the degree of mentalising. We need to assess the ability to incorporate inferences as to others’ mind into mental state inferences with tasks explicitly designed to do so (Conway et al. 2019b). Studies are needed that administer measures of social motivation, or which manipulate the instrumental benefits of mentalising, to see how these variables influence performance in tasks of spontaneous mentalising like the MASC. Furthermore, we need to develop more tasks that assess propensity, rather than ability to mentalise and administer these simultaneously to children with CP/HCU and comparison groups. Although we matched the groups on age, future studies may want to explore how mentalising changes as a function of age in children with CU. An important task for future research will be to consider the role of trauma and anxiety when assessing affective responses in children with CU as recent research has found differential responses to affective stimuli in children with high levels of trauma/anxiety and high levels of CU (Meffert et al. 2018). Finally, we only assessed boys and it will be important to see whether these difficulties extend to girls with CP/HCU.

## Conclusions

This study has the advantage of examining mentalising in three different ways which allows for refinement of understanding of mentalising in boys with CP/HCU. Overall, our findings suggest that boys with CP/HCU can successfully represent mental states when doing so does not require processing of complex information or when there is some potential instrumental advantage. They may find it easier or be more motivated to mentalise about peers or people their own age, as mentalising about peers typically has instrumental value. Although the capacity to mentalise is intact, which is necessary to be able to manipulate others, the reduced propensity to incorporate the mind of the other into mental state inference may allow CP/HCU boys to ignore the negative emotional consequences of their antisocial behaviour. This warrants further investigation with experimental tasks that vary the mind type and motivational context.

## Electronic supplementary material

ESM 1 (DOCX 409 kb)

ESM 2 (PDF 334 kb)
