# Supplementary material for: Thinking about Others’ Minds: Mental State Inference in Boys with Conduct Problems and Callous-Unemotional Traits
Source: J Abnorm Child Psychol. 2020 Jul 6;48(10):1279–90. doi: 10.1007/s10802-020-00664-1 (PMC7445196; doi:10.1007/s10802-020-00664-1)
Supplement: Supplementary file 1 — (DOCX 409 kb) [file 10802_2020_664_MOESM1_ESM.docx]

**The development of the Social Judgement Task (SJT): Assessing adolescents’ understanding of peer perception of negative interactions**

There are substantial individual differences in both judgements of social/moral appropriateness and level of disruptive behaviours in childhood and adolescence. Most children and adolescents have internalised societal norms, are concerned about how their behaviour is perceived by others, and do not typically engage in antisocial acts, however, a minority display problematic behaviour. We developed the Social Judgement Task (SJT); a new measure to assess adolescents’ self-reported likelihood of committing negative actions against a peer, and the degree to which they understand that other people see such actions as unacceptable.

**Task Development**

Using a similar format to Sharp et al. (2006), we presented a sample of typically-developing adolescents a series of ten stories accompanied by illustrative cartoons and asked them to imagine that they were the main character in the story. Several story options were generated, and the final stories were selected based upon their clarity and the potential for the action to happen in real life. The stories depicted negative, prosocial or neutral interactions with a peer. Half of the stories depicted negative interactions and these negative stories were the focus of the study. The prosocial and neutral stories were included to avoid presenting participants with solely negative content.

Instructions were as follows: ‘*You will be given 10 stories, each with a cartoon. Please read each story carefully. Each story involves interactions with kids around your own age and we would like you to imagine yourself as the main character in each story. After you have read the story, we would like you to guess what other kids might think of you if you behaved in the way that the story described. We will give you three possible choices and we want you to circle the answer that most closely describes what they would think of you.*’

Participants were presented with three response options for the positive and negative stories reflecting mutually exclusive categories: (i) belief that peers would find the behaviour acceptable; (ii) belief that peers would find the behaviour unacceptable; (iii) an emotionally naïve/neutral response. The two neutral stories had 3 response options: (i) a positive statement; (ii) a negative statement; (iii) and a neutral statement.

Participants were also asked how likely they were to commit the act in real life on a 1-5 point Likert scale. Stories were presented in a pseudo randomised order to ensure that no priming effects occurred. Figure 1. shows an example of a negative story.


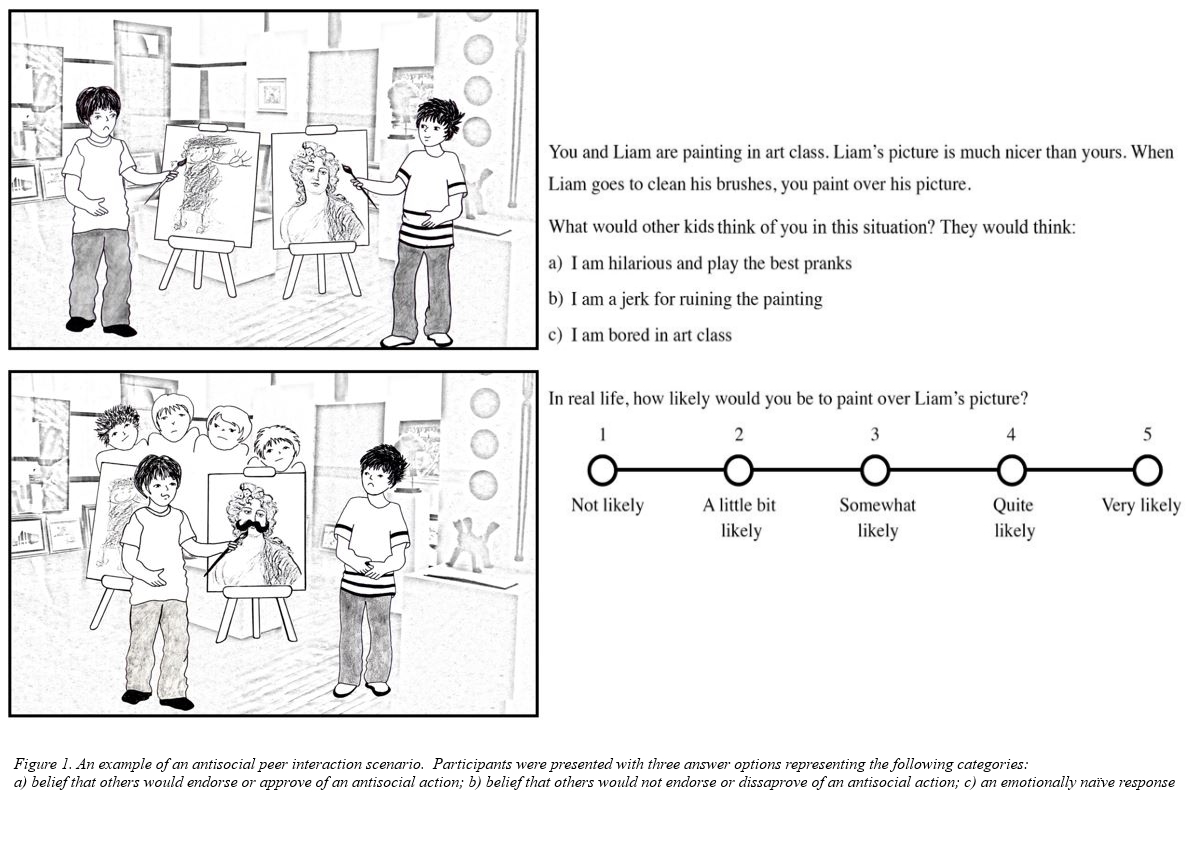


**Method**

**Participants**

Participants were recruited from a mainstream secondary school in Romford, Essex. The school was rated as ‘Outstanding’ by Ofsted school inspectors, with ninety percent of pupils achieving at or above expected level for their year. Nearly three-quarters of pupils at the school were from a minority ethnic background. Twenty percent of pupils at the school were eligible for free school meals.

One hundred and ninety-six adolescents participated in the research. Ten participants had more than 20% of the questionnaire data missing, indicating that the questionnaire had not been answered carefully. These participants were removed from all further analyses, leaving a final sample of N = 186. The age range of participants was 11-14 years old (M = 12.57, SD = 0.83; N = 2 did not disclose age). The sample was 53.8 % female (N = 100) and 45.2% male (N = 84); 1.1% of participants did not disclose their gender (N = 2). Ethnicity and socio-economic data were not collected as per request of the school.

Parental consent was obtained prior to testing. Questionnaires were administered in a classroom setting. A researcher read the instructions aloud to the class and informed all participants that their participation was voluntary. Participants completed the questionnaire independently and anonymously. No participant was identified to the researchers as having reading difficulties or special educational needs.

A random sample of 39 participants were re-assessed one week later to check reliability of the measure. Two participants were removed for having incomplete data at time one (T1) and one participant was removed due to incomplete data at time two (T2) leaving a final sample of N = 36 (20% of 186). The test-retest sample was 12-13 years old (M = 12.58, SD = 12.58) and 33.3% were male (N = 12).

**Measures**

In addition to the SJT, participants completed two additional questionnaires to assess the association between the SJT, callous-unemotional traits (CU) and conduct problems (CP). Brief measures were selected due to limited time for assessing participants in a school setting.

**Callous-unemotional (CU) subscale of the Antisocial Process Screening Device, Self-Report Version (APSD-SR; Frick & Hare, 2001).** Participants completed the six-item CU subscale of the ASPD-SR. Respondents were asked to rate their agreement with each statement on a 0-2 scale (0 = not true, 1 = sometimes true 3 = certainly true). The APSD-SR has been shown to have good psychometric properties (Munoz & Frick, 2007). We predicted that likelihood to commit the described negative interaction and the belief that others would find the negative interaction to be acceptable would be positively correlated with CU traits.

**Conduct problems (CP) subscale of the Strengths and Difficulties Questionnaire (SDQ; Goodman, 1997).** Participants completed the five-item CP subscale of the SDQ-SR. Respondents were asked to rate their agreement with each statement on a 0-2 scale (0 = not true, 1 = sometimes true, 2 = certainly true). The SDQ has been shown to have good psychometric properties (Goodman, 2001). We predicted that likelihood to commit the described negative interaction and the belief that others would find the negative interaction to be acceptable would be positively correlated with CP.

**Data analysis procedure**

All data were analysed using SPSS (version 21) unless stated otherwise.

**Defining SJT variables**

To investigate the likelihood of committing negative interactions against a peer, a sum of Likert scores on negative stories was computed (min = 5, max = 25).

To investigate the perception of others’ view of negative actions, a percentage of negative stories that participants thought peers would rate as acceptable was computed (range = 0-100%).

**Test-retest reliability**

To measure the stability of responses over time, a random subset of participants (20% of original sample) completed the SJT twice, one week apart. Wilcoxon signed rank test was used to examine the differences in likelihood scores between T1 and T2.

**Internal Consistency**

To establish internal consistency, Cronbach’s alpha was computed for negative and positive stories.

**Principal Components Analysis**

Principle components analysis was computed to determine if the likelihood scores for negative stories clustered together and the likelihood scores for positive stories clustered together.

**Construct validity**

Spearman’s correlations were computed to examine the associations between the two SJT variables (likelihood of committing negative interaction and belief that peers think negative interactions are acceptable), CU traits and CP. Spearman’s partial correlations were computed to assess the relationship between CU and the two SJT variables after adjusting for CP, and CP and the two SJT variables after adjusting for CU.

**Results**

**SJT variables**

As illustrated in Table 1, the majority of participants were unlikely to commit the negative interactions described in the stories. Six cases (3.2%) were missing one response so they were not included in this analysis.

Table 1. Likelihood of committing negative action

|  | Likert scores | | | |
| --- | --- | --- | --- | --- |
|  | 5 | 10-15 | 16 or more | Missing |
| Number of participants (percentage) | 140  (75.3%) | 28  (15%) | 12  (6.5%) | 6  (3.2%) |

5 = not likely; 10-15 = a little bit or somewhat likely; 16 or more = quite or very likely (min= 5, max = 25)

As illustrated in Table 2, the majority of participants believed that peers would have low levels of acceptance of negative actions, with over half the participants saying their peers would find none of the interactions to be acceptable. Six cases (3.2%) were missing one response so they were not included in this analysis.

Table 2. Number of negative stories participants thought their peers would rate as acceptable

|  | Number of negative stories peers would find acceptable | | | |
| --- | --- | --- | --- | --- |
|  | 0 | 1-2 | 3 or more | Missing |
| Number of participants (percentage) | 97  (52.2%) | 64  (34.4%) | 19  (10.2%) | 6  (3.2%) |

Out of a possible five negative stories

**Test-retest reliability**

A random sample of 39 participants completed the questionnaires twice, one week apart. As shown in Table 3, Wilcoxon signed rank test showed no difference between the mean likelihood scores for T1 and T2 on all items.

Table 3. Test-retest reliability: Wilcoxon signed rank test of likelihood scores at Time 1 and Time 2 (Interval = 7 days)

| Item | Sig. |
| --- | --- |
| Negative story ‘Art’ | 0.56 |
| Negative story ‘Crisps’ | 0.32 |
| Negative story ‘Fair’ | 0.11 |
| Negative story ‘Queue’ | 0.74 |
| Negative story ‘Treats’ | 0.66 |
| Positive story ‘Fiver’ | 0.59 |
| Positive story ‘Goal’ | 0.79 |
| Positive story ‘Phone’ | 0.41 |

Over 80% of participants selected the same response at T2 as they had selected at T1. One prosocial interaction story had only 63% agreement. With the exception of the one prosocial story with low agreement, the majority of participants were selecting responses that would reflect a belief that peers would think negative actions are unacceptable and prosocial actions are acceptable.

**Internal Consistency**

The five stories depicting a negative interaction had a high level of internal consistency, as determined by a Cronbach’s alpha of 0.81.

**Principle components analysis**

Principle components analysis (PCA) was computed on the likelihood scores for the five negative and three positive stories. The suitability of PCA was assessed prior to analysis. As shown in Table 4, the negative stories loaded on to one component and the positive stories loaded together on a second component.

Table 4. Principle components analysis: Likelihood scores for negative and positive stories

|  | Component | |
| --- | --- | --- |
|  | 1 | 2 |
| Negative story ‘Crisps’ | .83 |  |
| Negative story ‘Art’ | .80 |  |
| Negative story ‘Treats’ | .77 |  |
| Negative story ‘Queue’ | .73 |  |
| Negative story ‘Fair’ | .61 | -.36 |
| Positive story ‘Fiver’ |  | .75 |
| Positive story ‘Goal’ |  | .72 |
| Positive story ‘Phone’ | -.36 | .50 |

**Construct validity**

Spearman’s correlations were computed to examine the associations between the two SJT variables, CU traits, and CP. As shown in Table 5, the two SJT variables were positively correlated. The SJT variables were positively correlated with both CU traits and CP which is in line with hypotheses (see Table 5).

Table 5. Construct validity: Spearman's correlations between SJT variables, CU and CP

|  | | Belief peers think negative interactions are acceptable | Likelihood of committing negative interaction | CU | CP |
| --- | --- | --- | --- | --- | --- |
| Belief peers think negative interactions are acceptable | Correlation Coefficient | 1.000 | .380^**^ | .175^*^ | .235** |
|  | N | 180 | 178 | 180 | 177 |
| Likelihood of committing negative interaction | Correlation Coefficient | .380^**^ | 1.000 | .337^**^ | .333** |
|  | N | 178 | 180 | 180 | 177 |
| CU | Correlation Coefficient | .175^*^ | .337^**^ | 1.000 | .378** |
|  | N | 180 | 180 | 186 | 183 |
| CP | Correlation Coefficient | .235** | .333** | .378** | 1.000 |
|  | N | 177 | 177 | 183 | 183 |

*p < 0.05, **p < 0.001

Partial correlation analyses revealed that there was no longer a significant association between CU and belief that peers would find negative interactions to be acceptable after controlling for CP (*r* (174) = 0.096, *p* = 0.207). The association remained significant between CP and belief that peers would find negative interactions to be acceptable when controlling for CU (*r* (174) = 0.185, *p* = 0.014). Both CP (*r* (174) = 0.236, *p* = 0.002) and CU (*r* (174) = 0.241, *p* = 0.001) were significantly associated with the likelihood of committing a negative interaction, independent of the variance shared with the other dimension.

**Discussion**

This paper describes the development of the SJT, a measure to assess young people’s self-reported likelihood of committing negative actions against a peer and the degree to which they understand that other people see such actions as unacceptable. The SJT shows good test-retest reliability, internal consistency and construct validity. As expected from a typically developing sample of adolescents, the majority of participants were unlikely to engage in the described negative interactions with peers and believed that peers would find negative interactions to be unacceptable. However, there were individual differences in the self-reported likelihood of engaging in negative interactions and the belief that peers would find negative behaviour acceptable. The two SJT variables were positively correlated which indicates that if an individual does not understand that being disruptive is wrong, or indeed, thinks that this is an accepted way of behaving, he or she will be more likely to act disruptively. The two SJT variables were not perfectly correlated, indicating that there may be some individuals who know that peers find negative behaviour unacceptable but would choose to act negatively anyway. CU traits and CP were positively correlated with both the likelihood of acting negatively towards peers and belief that peers would find negative interactions to be acceptable, which indicates that the measure is able to characterise how the thinking patterns of adolescents with high levels of troubling traits and behaviours differ from their peers with lower levels of such traits and behaviours.

The SJT does not have clinical utility as a diagnostic tool but may be helpful in characterising some of the vulnerability in cognitive processing in adolescents with behaviour problems. Future research with the SJT will seek to assess clinical populations to see if different patterns of cognitive processing occur adolescents with more severe behavioural problems.

**References**

Sharp, C., Fonagy, P., & Goodyer, I.M. (2006). Imagining your child's mind: Psychosocial adjustment and mothers' ability to predict their children's attributional response styles. *British Journal of Developmental Psychology, 24,* 197-214.

Frick, P.J., & Hare, R. (2001). Antisocial process screening device. Toronto, Canada: Multi-Health Systems.

Munoz, L.C., & Frick, P.J. (2007). The reliability, stability, and predictive utility of the self-report version of the Antisocial Process Screening Device. *Scandinavian Journal of Psychology, 48,* 299-312.

Goodman, R. (1997). The Strengths and Difficulties Questionnaire: A Research Note. *Journal of Child Psychology and Psychiatry*, *38,* 581-586.

Goodman, R. (2001). Psychometric properties of the strengths and difficulties questionnaire. *Journal of the American Academy of Child and Adolescent Psychiatry, 40,* 1337-1345.
