# Supplementary material for: Thinking about Others’ Minds: Mental State Inference in Boys with Conduct Problems and Callous-Unemotional Traits
Source: J Abnorm Child Psychol. 2020 Jul 6;48(10):1279–90. doi: 10.1007/s10802-020-00664-1 (PMC7445196; doi:10.1007/s10802-020-00664-1)
Supplement: Supplementary file 2 — (PDF 334 kb) [file 10802_2020_664_MOESM2_ESM.pdf]

Journal of Abnormal Child Psychology

Thinking about others' minds: mental state inference in boys with conduct problems and callous-unemotional traits

Ruth Roberts[1]*, Eamon McCrory[1], Geoffrey Bird[2,3], Molly Sharp[1], Linda Roberts[4] & Essi Viding[1]

[1]Division of Psychology and Language Sciences, University College London, 26 Bedford Way, London WC1H 0AP, UK

[2]Department of Experimental Psychology, University of Oxford, Oxford, OX1 3PS, UK

[3]MRC Social, Genetic & Developmental Psychiatry Centre, Institute of Psychiatry, Psychology & Neuroscience, King's College London, De Crespigny Park, Denmark Hill, London, SE5 8AF, UK

[4]University of Manitoba, 66 Chancellors Cir, Winnipeg, Manitoba, R3T 2N2, Canada

*Corresponding author:

Ruth Roberts r.roberts@ucl.ac.uk

Supplementary Material Table 1. Spearman's Rho Correlations between MASC 'intentions' and child characteristics

		MASC 'intentions'	*Child group*	*CASI ADHD*	*CASI GAD*	*CASI MDE*	*BES cognitive*	*BES affective*	*IRI-PT*	*AQC*
MASC 'intentions'	*Correlation Coefficient*	1.00	**-.32****	**-.28***	**-.29****	-.15	**.40****	**.29****	.19	-.13
	Sig. (2-tailed)	.	.003	.011	.009	.181	.000	.009	.082	.239
	N	81	81	81	79	80	80	80	81	80
Child group	*Correlation Coefficient*	**-.32****	1.00	**.52****	**.48****	**.49****	**-.23***	**-.39****	**-.34****	-.04
	Sig. (2-tailed)	.003	.	.000	.000	.000	.040	.000	.002	.752
	N	81	81	81	79	80	80	80	81	80

MASC = Movie Assessment of Social Cognition ('intentions' items); CASI = Child and Adolescent Symptom Inventory; ADHD = Attention Deficit Hyperactivity Disorder; GAD = Generalised Anxiety Disorder; MDE = Major Depressive Episode; BES = Basic Empathy Scale (cognitive items / affective items); IRI-PT = Perspective taking subscale of the Interpersonal Reactivity Index; AQC = Alexithymia Questionnaire for Children
